# “We Have to Be Strong Ourselves”: Exploring the Support Needs of Informal Carers of Aboriginal and Torres Strait Islander People with Cancer

**DOI:** 10.3390/ijerph18147281

**Published:** 2021-07-07

**Authors:** Lorraine Bell, Kate Anderson, Afaf Girgis, Samar Aoun, Joan Cunningham, Claire E. Wakefield, Shaouli Shahid, Allan Ben Smith, Abbey Diaz, Daniel Lindsay, Adam Masa, Gail Garvey

**Affiliations:** 1 Menzies School of Health Research, Charles Darwin University, Casuarina, NT 0810, Australia; kate.anderson@menzies.edu.au (K.A.); joan.cunningham@menzies.edu.au (J.C.); abbey.diaz@menzies.edu.au (A.D.); daniel.lindsay@menzies.edu.au (D.L.); adam.masa94@googlemail.com (A.M.); gail.garvey@menzies.edu.au (G.G.); 2 Centre for Oncology Education and Research Translation (CONCERT), Ingham Institute for Applied Medical Research, South Western Sydney Clinical School, University of New South Wales, Liverpool, NSW 2170, Australia; afaf.girgis@unsw.edu.au (A.G.); ben.smith@unsw.edu.au (A.B.S.); 3 Public Health Palliative Care Unit, School of Psychology and Public Health, La Trobe University, Bundoora, VIC 3086, Australia; S.Aoun@latrobe.edu.au; 4 Perron Institute for Neurological and Translational Science, Nedlands, WA 6009, Australia; 5 Kids Cancer Centre, Sydney Children’s Hospital, Sydney, NSW 2031, Australia; c.wakefield@unsw.edu.au; 6 School of Women’s and Children’s Health, University of New South Wales, Sydney, NSW 2052, Australia; 7 Centre for Aboriginal Studies, Curtin University, Bentley, WA 6102, Australia; S.Shahid@curtin.edu.au

**Keywords:** family carers, cancer, Indigenous, unmet needs, cultural needs, qualitative

## Abstract

Informal carers provide an important role in supporting people with cancer. Aboriginal and Torres Strait Islander peoples experience higher cancer mortality than other Australians. To date, very little is known about the support needs of carers of Aboriginal and Torres Strait Islander adults with cancer. This article explored these needs through a qualitative study. Twenty-two semi-structured qualitative interviews and one focus group were conducted with carers of Aboriginal and Torres Strait Islander adults with cancer (*n* = 12) and Aboriginal and Torres Strait Islander cancer survivors (*n* = 15) from Queensland, Australia. Half of the carers interviewed were Aboriginal or Torres Strait Islander Australians. Interviews were transcribed, coded and thematically analysed following an interpretive phenomenological approach. Thematic analysis of carer and survivor interviews revealed four key themes relating to carers’ needs: managing multiple responsibilities; maintaining the carer’s own health and wellbeing; accessing practical support and information; and engaging with the health system. Within these overarching themes, multiple needs were identified including specific needs relevant for carers of Aboriginal and Torres Strait Islander peoples, such as advocating for the patient; accessing Indigenous support services and health workers; and ensuring that the cultural needs of the person are recognised and respected. Identifying the needs of informal carers of Aboriginal and Torres Strait Islander cancer patients will enable greater understanding of the support that carers require and inform the development of strategies to meet these areas of need.

## 1. Introduction

People with cancer often receive significant support from family members or friends who take on a role as an informal carer. Informal carers often juggle multiple responsibilities while caring for the person with cancer, including household and work duties, caring for other family members, adjusting to changing role expectations and managing their own health and wellbeing [1,2,3]. While carers play a significant role in caring for people with cancer, the focus within the healthcare setting largely remains on the patient’s experiences [4].

The importance of assessing the support needs of informal carers of people with cancer is well documented [3]. There are several carer needs assessment tools that can be used to offer insight into the specific needs of carers and the level of support they require to address each need [5,6,7,8,9,10,11,12,13,14,15]. Such information can assist in the prioritisation of services to best support and meet the needs of carers [16], which has been shown to increase carers’ capacity to provide care for patients [9,17]. 

Two key reviews of the support needs of partners and carers of people with cancer identified the following broad domains of need for carers: emotional and psychological support, spiritual needs, impact on daily activities and relationships, practical support, access to healthcare services and information needs [16,18]. Other literature has drawn attention to differences in carers’ needs and access to support services for different ethnic groups. For example, a study from the United States found that African-American carers use less support services and provide more hours of caregiving per week than Caucasian carers [19]. Further exploration of how Indigenous populations experience caregiving is required.

Recent evidence suggests that whilst carers of Indigenous people with cancer report some similar needs to carers of non-Indigenous patients, there are additional and specific needs requiring consideration [20,21]. A recent systematic review of the needs of carers of Indigenous people with cancer found that carers commonly reported feeling alienated, disoriented and disempowered in complex healthcare settings [21]. Carers described their role as mediators on behalf of Indigenous cancer patients between Western biomedical approaches to cancer care and Indigenous peoples’ holistic and family-centred views of health and wellbeing. This is likely to translate into different needs for carers of Indigenous peoples with cancer. If left unmet, these may underpin poorer mental health and quality of life and elevated carer burden for these carers [20].

In Australia, it is well documented that Aboriginal and Torres Strait Islander people with cancer have a unique set of supportive care needs [22,23]. Despite this, the needs of carers of this underserved patient population remain largely overlooked. This study aims to address this important gap by exploring the experiences of carers of Aboriginal and Torres Strait Islander people with cancer and identifying their support needs. In this paper, Aboriginal and Torres Strait Islander people are respectfully referred to as Indigenous Australians.

## 2. Methods

The study design was informed by Indigenous research methodologies that privileged the voices of Indigenous people and those with lived experience. The project was led by an Aboriginal researcher (GG) and approval was received by the Indigenous Steering Committee at the participating hospital.

This qualitative study gained an in-depth understanding of the lived experiences and needs associated with being a carer for an Indigenous person with cancer, using an interpretive phenomenological approach [24]. Semi-structured qualitative interviews and focus groups were conducted with informal carers of Indigenous cancer survivors, as well as with Indigenous cancer survivors to discuss their perception of their carer’s needs. The interview schedule was developed based on a priori understanding of the broader carer literature [3,16]. Topic areas covered included: activities undertaken by carers; challenges faced as a carer; engagement with health providers; support received and coping strategies; and reflection on the specific needs of carers of Indigenous peoples with cancer (see the Interview Schedule in Appendix A).

### 2.1. Participant Recruitment and Procedure

Carers were eligible to participate if they were 18 years or older and had been an informal carer for an Indigenous adult with cancer within the last 2 years. Informal carers are defined as family members or close friends who provide support to the person with cancer, such as taking the patient to appointments, assisting with medications etc. This does not include professional carers who are employed by an agency to provide care, as their needs and relationships with the client are not the same as informal carers. The carer did not have to be Indigenous themselves but had to be providing care for an Indigenous person. In this article, the term ‘cancer survivor’ is used to describe any person who has received a cancer diagnosis and is at any stage of their treatment or recovery. Cancer survivors were eligible to participate if they were 18 years or older, had received a cancer diagnosis in the last two years, identified as Indigenous and had at least one informal carer during their cancer experience.

Participants were recruited from a tertiary metropolitan hospital in Brisbane, Queensland, Australia in 2019. A staff member from the cancer centre (JT) identified Indigenous patients and invited the patient and their carer to the study. If interested, the patient and/or their carer gave permission for their contact details to be passed to a member of the research team (BA Indigenous researcher, LB non-Indigenous researcher). All participants were provided with a Participant Information Sheet and informed consent was obtained by the researcher. Study investigators also utilised their existing networks to identify and invite additional cancer survivors to participate in a focus group. 

Data was collected by four research team members: one Indigenous male (BA), one non-Indigenous male (AM) and two non-Indigenous females (LB, HMB). Interviewers had no clinical relationship with the cancer survivors or their carers and were not employed by the hospital. Participants were informed in the Participant Information Sheet that their participation would not affect their care. Prior to the interview starting, researchers introduced themselves and helped the participant feel at ease with a brief ‘social yarn’. [25]. Interview length ranged from 35 min to 123 min with carers and 13 min to 55 min with cancer survivors, while the focus group with cancer survivors lasted 91 min.

One-on-one interviews were conducted with carers and cancer survivors separately between June and November 2019, either face-to-face (*n* = 20) at a location convenient for the participant (e.g., hospital café, local library) or over telephone (*n* = 2). The focus group was held in August 2019 at a local community hall with cancer survivors (*n* = 5). The sample size of 27 participants, including 12 carers and 15 Indigenous cancer survivors, was deemed adequate to enable an in-depth understanding of the experience of being a carer of an Indigenous cancer survivor. Given the discrete scope of the research question, and the inclusion of both carers and cancer survivors to discuss carers’ needs, we were able to reach a level of data saturation with this sample. 

### 2.2. Ethics

This study was conducted in accordance with the NHMRC Guidelines for the Ethical Conduct in Aboriginal and Torres Strait Islander Health Research [26]. Ethical considerations for this study included ensuring that participants were aware that their participation was voluntary and that they could withdraw from the study at any time without explanation or penalty. The benefits of participating in this research was clearly conveyed to participants, and the methodologies described above allowed for the telling and sharing of stories in a culturally appropriate manner. Ethics approvals were received by the participating Queensland Hospital and Health Service, the Northern Territory Department of Health and Menzies School of Health Research Human Research Ethics Committee. Approval was also received by the Indigenous committee at the participating hospital, the Metro South Health Closing the Gap Steering Committee.

### 2.3. Data Analysis

Interviews and focus groups were audio-recorded and transcribed verbatim. Transcripts were deidentified and imported into NVivo12 software [27]. Three non-Indigenous researchers (LB, KA, AM) familiarised themselves with the data by independently reading a sample of transcripts and making notes on themes to inform the development of a preliminary coding framework. Transcripts were coded by LB and checked by AM using the preliminary coding framework. This was then discussed by LB, AM and KA to develop a final framework. This framework was reviewed by the Indigenous project leader (GG) to ensure cultural relevance. Following an interpretive phenomenological approach, interview transcripts were analysed line-by-line to inductively identify concepts and themes that reflected the experience of being a carer of an Indigenous person with cancer [24]. Patterns within the data were identified until data saturation was achieved.

## 3. Results

Twenty-seven people participated (12 carers of Indigenous cancer survivors and 15 Indigenous cancer survivors). An additional twenty-five people (9 carers and 16 cancer survivors) either declined or did not respond when invited to participate. Reasons for declining were not always provided, but some people indicated they were unable to participate due to time constraints and competing health priorities. Participant characteristics are provided in Table 1. Carers were on average, 48 years old (range 19–67 years; 58% female) and had been in a caring role for a median of eight months (range 3 months to 10 years). Half the carers were Indigenous, and half were a cancer survivor’s spouse. Cancer survivors were on average, 53 years old (range 27–71 years; 53% female) and were diagnosed with a range of cancer types. Almost half (47%) were receiving treatment at the time of the interview, with chemotherapy being the most common treatment (73%).

Thematic analysis identified four themes relating to carer’s needs: managing multiple responsibilities; maintaining carers’ own health and wellbeing; accessing practical support and information; and engaging with the health system. Within these overarching themes, needs were expressed in terms of the *general* experience of being a carer for a person with cancer, as well as needs specific to the experience of caring for an Indigenous person. There was congruence between themes identified by both carers and cancer survivors, and these are presented together below. Table 2 summarises the needs identified within each theme and classifies the need as general or Indigenous-specific. 

### 3.1. Managing Multiple Responsibilities

Central within carers’ narratives were accounts of the challenges associated with managing multiple new and shifting roles and responsibilities within the family, which often led to feelings of distress and fatigue. These responsibilities included carers having to juggle their new and existing roles, which for some included making end-of-life preparations.

#### 3.1.1. New Responsibilities 

Carers described grappling with a range of unfamiliar and challenging roles in the provision of practical, emotional, physical and communication support to cancer survivors. Many of these new roles were particularly fraught for carers, as they had little preparation or training. 

New practical roles, such as accompanying the survivor to medical appointments and additional household responsibilities, were time consuming and challenging for some carers. Carers also provided emotional and healthcare support, including providing help with giving medications. For some, this responsibility felt overwhelming, with carers reporting a need for more information and training. 

Communication within and between family members, friends and healthcare providers was another responsibility reported by carers. At times, carers were required to act as a mediator, gatekeeper or advocate for the cancer patient.

*“So Mum was a health professional anyway, so she knew the language, she knew what was being said. What she needed was the advocates because she was quite humble. She wouldn’t ask those hard questions. (…) had Mum not had the assertive carers around her, my brothers and my sister, I think that would’ve been problematic down the track...”*

*(C08; Indigenous carer)*

The role carers played in managing family and friends was greatly appreciated by survivors, particularly when large family and community groups were involved:

*“…everyone would just bombard me with phone calls and text messages and she [my carer] was kind of like to everyone, she’s going to call you back later (…) like my secretary or something. When I wasn’t really, you know, feeling up to having all these different conversations with everyone…”*

*(S09; Indigenous cancer survivor)*

To help manage the flow of communication, some carers found it useful to use social media and online groups to stay connected and keep family involved in the process.

The carer’s role included having difficult conversations with family members about the patient’s cancer diagnosis and prognosis. Some carers reported this was particularly difficult, especially when younger children were involved.

*“The hardest part I think, looking back, was always telling my kids. That was hard, how do I do that? Even when [the patient] went into ICU and they were just like basically saying he is not doing too well, but how do I tell the kids that?”*

*(C03; non-Indigenous carer)*

This participant further explains she would have appreciated help or guidance to know how best to communicate this news with family members *“Just someone … to sit down and explain what is happening to a 5-year-old or to a 16-year-old.” (C03; non-Indigenous carer).*

#### 3.1.2. Shifting Roles within Families

For many Indigenous families, there was an unspoken acceptance of taking on the role of carer for your family members *“… being Aboriginal, we look after our mob, ourselves, our family and that’s the way I was brought up” (C04; Indigenous carer).*

At times, taking on the role of carer required a shift in usual family dynamics, such as children caring for their parents. For larger families, there were different roles that each family member would take on. This included some taking a ‘coordinator’ role, while others focused on caring for the primary carers and providing them with respite. When multiple people were involved in caregiving, communication and organisation were particularly important. 

#### 3.1.3. Juggling Existing Roles

As well as new and shifting roles, carers also described juggling existing responsibilities, including work, childcare and household duties. Support to maintain work responsibilities was a key need identified by many participants. While many carers were able to negotiate leave arrangements or flexible work hours, they described it as exhausting to manage both *“I’m still working and it’s just hard going backwards and forward to the hospital because we’ve been going there twice a week.” (C11; non-Indigenous carer).*

It was also emotionally challenging for some carers to leave their job due to caring responsibilities.

*“… the hardest part was accepting that I wasn’t going to be able to work. I thought that I would be able to juggle both. On his good days we would do the hospital and I would come back to [work] and no, it didn’t work”*

*(C02; non-Indigenous carer)*

#### 3.1.4. Making End-of-Life Preparations

In cases where preparation for death was necessary, carers had an important role to play. While end-of-life preparations could be upsetting for some carers, it was also recognised as a time of great significance. One participant described how, in Aboriginal culture, the people responsible for caring have the honour of learning the stories of the person with cancer to pass on to future generations.

*“In terms of preparing for death, what we do from a cultural perspective is we revisit places of my mother’s—so my mother would take us to places of her childhood and she’d tell the stories of that place, so we can continue the stories on. (…) And what happens is the carers who are around her during these journeys and trips become knowledge keepers. (…) Walking with someone who is passing during this period is an extremely privileged position, as a carer, as a family member, all that sort of stuff.”*

*(C08; Indigenous carer)*

### 3.2. Maintaining Carers’ Own Health and Wellbeing

A key issue for carers was the importance of trying to maintain their own health and wellbeing while also tending to the needs of the person they are caring for.

#### 3.2.1. Finding Time for Self 

Carers identified a need for ‘time-out’ to keep themselves healthy and strong. However, they expressed difficulty finding this time. One carer commented:

*“I don’t get to really relax. It’s finding time for that which is hard or I stay up late at night so I can watch a movie… But then it means I’m staying up really late and getting little sleep, because I have to be up in the mornings so I can help her again. So, it’s like changing around my schedule or whatever, just so I can have that time. I find it hard to deal when I don’t get time to do that.”*

*(C06; Indigenous carer)*

The need for carers to have time off was also recognised by Indigenous cancer survivors, who acknowledged the physical and emotional toll that their carers experienced.

#### 3.2.2. Psychological and Emotional Support

The need for psychological and emotional support emerged as a key consideration for carers. It was common for carers to feel overwhelmed and experience anxiety, stress and fatigue. There was also high stress around uncertainty and change, with many participants describing the “*not knowing*” during the treatment phase as very difficult. This was identified as a time when support was especially required, as one participant explains:

*“… that’s the time when you’re more in limbo, because there’s no support (…) And that’s what I would love to change for more people, not just doctors, but just the psychologist to come and—because it’s very emotional—it’s such an emotional rollercoaster for him and for me, because I don’t know what to say or don’t know how to make it better or to help him through. And I spent many days crying, yeah, I did. It was awful”*

*(C12; Indigenous carer)*

There was also an unspoken, self-imposed expectation for carers to stay emotionally strong, as one daughter explained when looking after her mother, *“It was more that you were going to bed and you’re thinking about things, that’s when I started the crying and the, ‘oh my God please don’t take her’, but I was strong around her because I didn’t want to get her upset” (C01; Indigenous carer)*

Some carers also expressed the need for regular support throughout their care journey, especially when they were caring for people at home and at times felt forgotten. One participant noted she would have appreciated regular phone calls from health workers to ‘check-in’ and see how she was feeling. Participants also reported receiving emotional support in other ways; many benefited from talking to other carers and sharing their experiences. Some carers also mentioned the emotional support they received through their faith and spirituality. 

#### 3.2.3. Staying Healthy and Maintaining Social Connections

The social aspect of health and wellbeing was often unfilled in the carers. Some carers felt socially isolated because they could not leave the house or participate in their usual social activities. Carers felt pressure to stay on top of their own health in order to care for the patient and avoid passing on sickness, especially when caring for someone with a weakened immune system. As one carer explained, *“we have to be strong ourselves. We can’t afford to be sick... If the carer is in the home, they can’t be sick” (C07; non-Indigenous carer).* In some cases, carers made lifestyle changes, such as avoiding alcohol, so they could fulfil their caring duties. 

The need for support services for carers’ own wellbeing was also raised by cancer survivors:

*“I think if there were more services willing to support the carers as well. Not just the patient but the carer as well because they need it because it’s mentally and physically—if they don’t have the support as well, it can really take its toll on them (…) and then they forget about their own health, you know.”*

*(S03; Indigenous cancer survivor)*

### 3.3. Accessing Practical Support and Information

Participants described issues with awareness of, and access to, practical support services, highlighting the need for greater information about the services available. 

#### 3.3.1. Practical and Financial Support Needs

Practical support needs were identified as a key consideration for carers, especially financial assistance with costs associated with hospital visits (transport, parking etc.) and the cost of medication: *“Financial, is the hardest thing… You have got to have that money flowing through to pay the bills, the bills don’t stop because you get sick. If everything wasn’t so expensive it would be good” (C13; non-Indigenous carer).* Some carers suggested practical ways that could help with the financial burden, such as a fuel voucher or parking card. 

Most services were focused on the patient’s needs, but there were also some practical forms of assistance available for carers, such as financial assistance or help with household duties (e.g., cleaning, mowing). However, many participants reported a lack of awareness of services available for carers and how to access them. Seeking carers’ financial assistance via the Australian Government Social Services (Centrelink) was also challenging, as carers were not sure how to apply for this assistance: *“… I didn’t know how to go about applying for a carer [allowance]; I still don’t.” (C12; Indigenous carer).* Some carers and cancer survivors found it difficult to find out what they were entitled to, *“I believe there was a lot of stuff we could have done on Centrelink that we didn’t do, that we didn’t find out about.” (S14; Indigenous cancer survivor)*

An additional challenge was reported for carers who did not feel comfortable asking for help, and this impacted on their awareness and access to services. 

#### 3.3.2. Getting Appropriate Health Information

Carers described feeling inadequately informed about cancer generally and the medical aspects of caring for a person with cancer. Personal preference dictated the level and type of information carers wanted to have about their patient’s cancer diagnosis and treatment. Many described an aversion to looking up medical information online as this would often present worst-case scenarios. It was also important to ensure the information was understood by the carer and patient. There was limited information available specifically for carers, with most of the information provided by the hospital targeted towards the patient. As one participant explained, *“…I didn’t really get any flyers or things like—you can go and talk to these people who are carers. Nothing like that.” (C05; Indigenous carer)*

### 3.4. Engaging with the Health System

Engagement with the health system encompassed coordination between services, interactions with and between health professionals, navigating and understanding the health system and the need for culturally-safe care. 

#### 3.4.1. Coordination between Services

Carers were often required to fulfil the role of mediator between the patient and their health providers. When there was lack of coordination between services, this commonly resulted in frustration and stress for the carer. The father of one patient described his frustration when doctors from a regional and a metropolitan hospital did not communicate, resulting in delays in his daughter’s treatment:

*“And then she was stuck up there… it was about four days… And it broke me, because one doctor wouldn’t talk to this other doctor and, you know, it was stressing me out a bit there at one stage, you know”*

*(C04; Indigenous carer)*

#### 3.4.2. Interaction with Health Professionals

Participants reported that there were times when they did not feel their role as carer was recognised or respected by health providers.

*“But I just find as a carer you’re not treated really respectfully. They kind of treat you like you’re just there to dress them and bathe them. They don’t seem to realise you’re with the ride in the journey, and sometimes my husband wouldn’t know what tablets he is taking, and I’d tell them that, but they’d kind of looking at you like you’re just coming along, you don’t know.”*

*(C07; non-Indigenous carer)*

The need for better identification of carers in patient records was also flagged, so no assumptions are made about who is ‘the carer’.

*“…the first thing that they ask you is for your details, and then your partner or whatever. So I had to keep reminding them that Dad was the one that was going to be with me all the time and giving them his number and saying he’s the one to call…”*

*(S02; Indigenous patient)*

#### 3.4.3. Navigation and Understanding the System

Navigating a complex health system was identified as an issue for some participants. Carers reported confusion around where and who they could go to for assistance. Participants who had access to the Indigenous Liaison Officer found this service helpful, as they provided information about services and support and made them feel welcome.

*“…the Indigenous Support Officer here (…) has organised transport vouchers to get us home, which is absolutely fantastic. (…) And they have always got their room. It has got tea and coffee and the television….”*

*(C02; non-Indigenous carer)*

However, others noted they had limited interaction with an Indigenous Liaison Officer or little understanding of their role. Yet, there was an expressed need to have a dedicated person who could be there to help explain the issues or considerations for Indigenous families.

Being able to access local services and engage with staff who have a personal relationship with the family was considered important for Indigenous families experiencing cancer. Some participants also found it helpful to receive support from their local Indigenous community services.

*“It’s not as hard as I thought it would be, but I think that’s because we get help from [the local Indigenous Health Service]. She gets free medical help a lot and through that she [has access to a gym service]”*

*(C06; Indigenous carer)*

#### 3.4.4. Accessing Culturally-Safe Care

Access to culturally-safe care for the patient was identified as a key factor that impacted on the experience and coping of carers. Some carers felt that the cultural needs of patients were not well respected and accommodated by the healthcare team. This was exemplified in one carer’s experience with end-of-life care for his mother and her wish to die at home, near her birthplace. The family felt hostility and lack of support from the healthcare team to facilitate this request and this impacted on the wellbeing of not only the patient, but all the family members.

*“We can deal with the limited support or lack of information or the resources (…) But when it came to palliative care, the hospice conversation, all that sort of stuff, that’s when things got really prickly.”*

*(C08; Indigenous carer)*

## 4. Discussion

This qualitative study fills a gap in the literature on the needs of carers of Indigenous cancer survivors in Australia and offers insight into the unique considerations for this carer group. As well as identifying unique needs, such as advocating for cultural requirements, this study also corroborated the areas of support that have been previously reported for carers of non-Indigenous cancer survivors. This study’s findings, such as the understanding the cultural needs of the cancer patient, align with the identified needs of family carers for cancer patients living in other countries [28]. Further, as found with other studies of carers of cancer survivors, carer needs relate to both providing care for the cancer survivor as well as the carer’s own personal health and wellbeing. This latter aspect of caregiving can be overlooked in the health setting, yet it is important for support systems to be in place that address the dual aspects of caregiving: acknowledging the carer as a co-worker in supporting the person with cancer and as a co-client in the healthcare setting [29,30]. 

Similar to previous studies with carers of non-Indigenous cancer survivors, we found that carers are required to fulfil their role of carer alongside many other responsibilities in their life, such as work or study commitments and caring for other family members [17,31,32]. This is a source of stress for many carers and can contribute to carer burden [33]. To alleviate stress and anxiety, carers in the current study expressed a need for emotional support across different stages of the cancer journey, especially immediately after diagnosis and during periods of prognostic uncertainty, either from allied health workers or other carers who had similar experiences [4,34]. Other common needs identified in the current and previous research related to difficulties accessing practical support (e.g., financial assistance), information needs and assistance navigating the health system [35]. 

There are additional aspects of caring for an Indigenous person with cancer that lead to unique considerations for the carer. This study found the type of needs and support required by carers is influenced by the diverse cultural beliefs, practices and values identified by Indigenous cancer survivors [36]. For example, participants in the current study identified the need for carers to advocate for the cultural needs of the patient. This role of ‘cultural broker’ has also been reported in other studies of Indigenous carers, with carers being responsible for facilitating communication between the cancer survivor and health professionals [37]. Similar to our findings, advocating for the patients’ cultural needs has previously been reported as especially important for end-of-life care and maintaining strong connections to Country [38]. To assist the carer in fulfilling this cultural role, it is necessary to ensure culturally safe systems are in place with opportunities to speak about cultural needs with health professionals [21].

Carers also played an important role as communicator, not only with health professionals, but also with family members. With many Indigenous people having large family networks, the challenge of communicating with multiple family members can be heightened. These communication challenges were also noted in the recent international systematic review of the needs of carers of Indigenous cancer survivors [21]. Systems need to be in place to support communication within the family unit. This may include, for example, strategies to assist with keeping multiple family members informed (e.g., group messaging services) as well as information and practical help for talking to younger family members about cancer. Recognising the carer’s communication needs within their broader family context is important for providing a family-centred approach to care, as reflected in other studies of carers of Indigenous cancer survivors [21]. Other studies of Indigenous cancer survivors have also identified the role of multiple family members taking on caring responsibilities [39]. This was less prominent in our sample with caring commonly falling to one main carer, such as the spouse, yet this was still within the context of broader family involvement and reflects a need for family-focused care rather than an individual patient-model [21]. 

Participants in the current study expressed needs regarding information and support services to help the carer navigate the health system and know what avenues of help were available. In our study it was recognised that Indigenous Liaison Officers can play an important role in negotiating complex health systems and making people feel welcome, yet only when people are aware of what support and guidance they can provide. The importance of Indigenous health workers has also been reported in other Australian studies [38,40]. It is important for culturally-appropriate information to be provided that is targeted to an appropriate level of health literacy [21]. To facilitate care at home, there is a need for greater information and training for carers, as is also found in other studies [35,41]. Some carers are reluctant to ask for help, so it is important for health services to employ a proactive and targeted approach to providing information to carers to suit their particular needs and regular check-ins by health professionals to assess any changes in needs over time. These check-ins should extend beyond the hospital setting, to consider the needs of people who are providing care at home. 

Some additional issues were raised in previous studies of Indigenous carers needs that were not identified in our mainly urban sample [20,21]. For example, needs around living away from home and associated accommodation expenses were not identified in our study, but may be an important consideration for people living outside of urban centres [40,42] and can add to financial costs for people who live in rural areas [43]. A recent study of Indigenous cancer patients’ access to cancer services in the Northern Territory, found that dislocation from home and challenges associated with language, accommodation, transport and finance posed major barriers to accessing treatment [40]. Thus, it is important to take into consideration the geographic context and language challenges that may be experienced by some carers of Indigenous people with cancer.

To assist carers of Indigenous people with cancer in getting the relevant support to meet their needs, findings from this study could inform the development of a needs assessment tool for carers. Existing tools for carers of people with cancer have had limited focus on cultural needs. The Needs Assessment Tool for Carers of People with Advanced Cancer (NAT-C) is, to our knowledge, the only tool that includes an item related to culture, stating “the illness and its effects are challenging because of my culture, or the other person’s culture” [9]. However, this single item does not address the depth of experience and needs of carers of Indigenous people with cancer, as illustrated in this study.

## 5. Strengths and Limitations

This paper addresses an important gap in our knowledge of the needs of carers of Indigenous cancer survivors in Australia. While it has been documented that Indigenous cancer patients experience unique support needs [22], limited research had focused on the needs of carers of this patient group. The new insights proffered by this study advance our understanding of the support needs of cancer carers, in general, as well as the unique needs of caring for an Indigenous person with cancer.

Whilst this study makes an important contribution to the evidence regarding the needs of caregivers, there were several limitations to this study. A broader sample may have identified additional needs relevant for the experience of carers of Indigenous people with cancer. Most participants were recruited from a metropolitan hospital and the majority of carers and cancer survivors lived within a 30 kilometre radius from the hospital, thus the views expressed and themes identified in this study represent the experience of mainly urban Indigenous cancer patients and their carers. This may not reflect the experience of people from other regional or remote areas of the country. In addition, most carers in this study were caring for people in the treatment or recovery phase, and thus their needs reflect that aspect of caring. Only one carer in this study had experienced end-of-life care and bereavement. Future research should also aim to collect additional data on the carer, including their occupation and hours spent working, and where possible more clinical information about the patient to further understand the burdens that may influence the level of need for carers of cancer survivors. It is also worth noting that many carers in this study spoke about their needs retrospectively, rather than reporting on their *current* experience of need. The type of support perceived necessary on reflection, may be different to the type of support accessed during these heightened moments of need. A more diverse sample involving people living in regional and remote areas of Australia and caring for people at different stages of the cancer care journey may provide additional insight into the experience of carers of Indigenous cancer patients.

## 6. Conclusions

This qualitative investigation of the needs of informal carers of Indigenous people with cancer has shown that while some identified needs are common among other carer groups (e.g., a need for emotional support), there are needs that are unmet and specific to the experience of caring for Indigenous Australians with cancer. Our findings highlight that health professionals need to be attentive to needs of carers of Indigenous cancer survivors. Having a tailored assessment tool that captures these areas of need will assist health professionals to systematically identify and address these needs.

## Figures and Tables

**Table 1:** Demographic and clinical characteristics of participants (*N* = 27).

Carer (*n* = 12)		Cancer Survivor (*n* = 15)	
Sex		Sex	
Male	5	Male	7
Female	7	Female	8
Age		Age	
18–24 years old	1	18–24 years old	0
25–34 years old	1	25–34 years old	3
35–44 years old	1	35–44 years old	1
45–54 years old	5	45–54 years old	3
55–64 years old	2	55–64 years old	5
65–74 years old	2	65–74 years old	3
Indigenous Status		Indigenous Status	
Indigenous	6	Indigenous	15
Non-Indigenous	6	Non-Indigenous	0
Time in Carer Role		Number of Carers	
3–6 months	4	One Main Carer	10
7–11 months	3	More than One Carer	5
1–2 years	3	Currently Receiving Treatment	
3–5 years	0	Yes	7
6+ years	2	No	8
Carer’s Relationship to Patient		Patient’s Relationship to Carer	
Wife	4	Wife	2
Husband	2	Husband	5
Sister	1	Sister	2
Son	1	Brother	1
Daughter	2	Daughter	1
Father	1	Mother	3
Friend	1	Friend	3
Distance home to treating hospital		Cancer Diagnosis	
10–30 km	8	Blood Cancer	4
30–50 km	1	Lung	3
70–1000 km	1	Pancreatic	2
1000–2000 km	2	Breast	2
Area of Usual Residence		Brain	1
Metropolitan	10	Thymoma	1
Inner Regional	1	Colorectal	1
Outer Regional	1	Nasopharyngeal	1
		Treatment Type	
		Chemotherapy	11
		Radiation Therapy	7
		Surgery	7
		Hormone Therapy	2
		Immunotherapy	1
		Area of Usual Residence	
		Metropolitan	12
		Inner Regional	3

**Table 2:** Summary of themes, sub-themes and needs for carers of Indigenous cancer patients.

Theme	Sub Themes	General Carer Needs	Indigenous-Specific Needs
Managing multiple responsibilities	○ New responsibilities ○ Shifting roles within families ○ Juggling existing roles ○ Making end-of-life preparations	Providing practical support to the person with cancer (e.g., attending appointments, administering medications) Providing emotional support to the patient Effectively communicating with, informing and updating family and friends Managing family and friends visiting or contacting the patient Emotional or practical assistance with balancing work and caring responsibilities	Advocating for patient and communicating with healthcare providers about patient’s cultural needs Preparing for end-of-life and passing on to Country Helping the patient to pass on stories to future generations
Maintaining carer’s own health and wellbeing	○ Finding time for self ○ Psychological and emotional support ○ Staying healthy and maintaining social connections	Staying on top of carer’s own health needs Staying socially connected with family and friends Finding time for self Staying emotionally strong Emotional support from healthcare team Support from faith and spirituality Emotional support from other carers	
Accessing practical support and information	○ Practical and financial support needs ○ Getting appropriate health information	Practical support needs (finance, transport, etc.) Accessing medical training and education for care at home Awareness of services for carer and/or patient Accessing government services for carer and/or patient Accessing services to support the patient and/or carer Getting information about cancer Information specifically for carers	Finding information about Indigenous services to support the patient and/or carer
Engaging with the health system	○ Improved coordination between services ○ Interaction with health professionals ○ Navigating and understanding the system ○ Accessing culturally-safe care	Ensuring the healthcare team are communicating with each other/sharing necessary information about the patient Feeling respected by the healthcare team Identification of carers in patient records Knowing who does what and who to approach for support	Accessing support from an Indigenous Liaison Officer Feeling welcome and comfortable in the health service Accessing local Indigenous community health services Having cultural needs recognised and respected

## Data Availability

The data used in this study are not available due to privacy and confidentially restrictions.

## Appendix A

**Interview Schedule for Carers**

1. Can you tell me about your experiences as a carer?
2. Sometimes people will be a carer for someone with cancer for part of their cancer journey, [referring to cancer journey diagram] like just when they were diagnosed, or having treatment, or recovering after treatment or during palliative care. Which parts of the journey were you involved in?
3. Did/do you share the care of [PATIENT NAME] with anyone?
4. Can you tell me about the sorts of things that you have done or are doing in your role as a carer?
5. Carers can take on a lot of responsibilities when looking after someone with cancer. I’m interested in hearing about any difficulties you personally have had. What were some of the challenges you faced or are still facing?
6. Caregivers sometimes balance different roles and duties at the same time—like family duties, working and caring for other people. Could you tell me about any other roles you had while you were [PATIENT NAME] carer?
7. What were your experiences dealing with care providers and health professionals for [PATIENT NAME]? What sort of things would you have liked support with when you were dealing with care providers for [PATIENT NAME]?
8. What sort of information did you receive from the health professionals?
9. How much information would you prefer to get from the hospital about [PATIENT NAME’S] cancer, treatment, and their future outlook?
10. What sort of help or support did you receive for the challenges you mentioned above as a carer, if any? From who?
11. How did you hear about where and who you can get support from?
12. Were there any times you felt you weren’t coping while being a carer?
13. Are there things that have helped you to cope as a carer?
14. As a carer, were there things that were meant to be helpful but were unhelpful, unnecessary or annoying?
15. What advice would you give to other carers of Indigenous patients with cancer to help them cope or access support?
16. Reflecting on your experience as a carer now, do you think there have been any good things about being a carer, despite some of the difficulties involved?
17. Thank you for sharing your personal experiences with me. Is there anything else that you would like to add to our discussion of the needs of people who care for Indigenous people with cancer?

**Interview Schedule for Cancer Survivors**

1. Can you tell me about your carer/s?
2. Can you tell me about the sorts of things your carer/s do/does/did in their role as a carer?
3. What sorts of things would you have liked your carer/s to be able to do?
4. What sort of things do you think would have made it easier for your carer/s to support you?
5. Were you happy with how much or little your cancer care providers (e.g., nurse or doctor) included your carer/s in discussion and consultations?
6. Do you think there have been any good things that came out of these experiences for you and your carer?
7. Thank you for sharing your personal experiences with me. Is there anything else that you would like to add to our discussion of the needs of people who care for Indigenous people with cancer?
